# A 20-Year Longitudinal Study of Homelessness Among 333 Psychiatric Patients in Portugal: From Initial Episodes to Chronic Trajectories

**DOI:** 10.1177/00207640261468246

**Published:** 2026-07-29

**Authors:** Joana Henriques-Calado, Nathalia Garcia, João Gama-Marques

**Affiliations:** 1 Centro de Investigação em Ciência Psicológica (CICPSI), Faculdade de Psicologia, Universidade de Lisboa (FPUL), Portugal, European Union; 2 Faculdade de Psicologia, Universidade de Lisboa (FPUL), Portugal, European Union; 3 Consulta de Esquizofrenia Resistente (CER), and Homeless Outreach Psychiatric Engagement for Lisboa (HOPE 4 Lisboa), Hospital de Júlio de Matos (HJM), Unidade Local de Saúde de São José (ULSSJ), Centro Clínico Académico de Lisboa (CCAL), Portugal, European Union; 4 Clínica Universitária de Psiquiatra e Psicologia Médica (CUPPM), and PsyLab Instituto de Saúde Ambiental (ISAMB), Faculdade de Medicina, Universidade de Lisboa (FMUL), Centro Académico de Medicina de Lisboa (CAML), Portugal, European Union

**Keywords:** homelessness, mortality, survival, comorbidity, psychiatry, longitudinal trajectories

## Abstract

**Background:**

Homelessness is closely related to psychiatric disorders, substance abuse, and premature mortality; however, in-depth analyses of long-term trajectories are lacking.

**Aims:**

The objective was to analyze psychiatric diagnoses, clinical patterns and survival outcomes of homelessness trajectories during a 20-year follow-up.

**Method:**

A retrospective sample of 333 psychiatric patients experiencing homelessness in Portugal. Cox survival analyses defined the cohorts: Recent (⩽1 year; *n* = 92), Early-stage (1–5 years; *n* = 52), Prolonged (5–15 years; *n* = 93), and Persistent (>15 years; *n* = 96). Analyses included analysis of variance (ANOVA), Chi-square tests, and logistic regression.

**Results:**

Mortality was 31.2%, decreasing after 18 years of homelessness, indicating a survivor effect. Recent cohort and younger patients, and those with alcohol misuse, anxiety, and personality disorders had minimal hospitalization. Early-stage and Prolonged groups had the highest mortality (50%), linked to alcohol-related disorders and organic psychosis, mainly in Prolonged. Persistent cohort, with the longest psychiatric follow-up (18.8 years), more hospitalizations, and higher comorbidity with drug use and intellectual disability, had the lowest mortality (14.6%) and the highest mean age at death (67.3 years). Unspecified psychosis, substance use, and intellectual disability were the key predictors of Persistent group. Alcohol use, psychosis, and personality disorders influenced survival varying by cohort.

**Conclusions:**

Patient psychiatric homelessness cohorts have heterogeneous trajectories for clinical, sociodemographic, service use, and survival. Early and mid-stages of homelessness show higher mortality. Findings highlight the Persistent cohort as long-term homelessness survivors. It is stressed that the duration of homelessness is a critical determinant of diagnostic patterns, comorbidities, and survival outcomes, which require differentiated psychiatric and social interventions tailored to cohort-specific risks among people experiencing homelessness.

## Introduction

Homelessness represents a critical concern in contemporary psychiatry due to its widespread effects on the manifestation of psychopathology, overall clinical and service functioning, and long-term prognostic trajectories (Bassuk et al., 1984; Herrera-Imbroda et al., 2023; Nilsson et al., 2024). Misused terminology weakens diagnostic precision, perpetuates stigma, and hinders appropriate psychiatric care (Bento & Barreto, 2002; Gama Marques, 2024; Gans, 2010).

The psychiatric burden among people experiencing homelessness (PEH) stems from interactions between individual vulnerabilities, such as childhood adversity, substance use, and psychiatric disorders (Nilsson et al., 2019; Shelton et al., 2009), and structural determinants, including socioeconomic exclusion and unstable access to healthcare and housing (Barreto & Cockersell, 2024; Cooper & McCulloch, 2023; Folsom et al., 2005). Epidemiological studies consistently report markedly higher rates of schizophrenia spectrum, mood, substance use, and especially personality disorders (Ball et al., 2005; Barry et al., 2024; Dell et al., 2023; Fazel et al., 2008; Gama Marques et al., 2024; Gutwinski et al., 2021; Henriques-Calado & Gama Marques, 2024; Kotamarti et al., 2021; Monteiro Fernandes et al., 2022; Neves Horácio et al., 2023; Salavera et al., 2013). Prolonged or early-onset homelessness worsens psychiatric decompensation, fosters maladaptive coping, increases reliance on fragmented crisis-orientated systems, and increases mortality, in a clinical profile inherently dynamic (Brown et al., 2022; Kuhn & Culhane, 1998; Leonori et al., 2000; North et al., 2004).

In Portugal, homelessness has become an increasingly visible public health and social problem. Recent national data report approximately 14.476 people experiencing homelessness, including around 9.403 people in roofless situations and 5.073 in houseless conditions, reflecting the structural complexity of the phenomenon (Estratégia Nacional para a Integração de Pessoas em Situação de Sem-Abrigo (ENIPSSA), 2024). The profile is predominantly male, of Portuguese nationality, single, between 45 and 64 years old, with 6 or 9 years of schooling. A substantial proportion of cases are concentrated in urban areas, particularly in Lisboa, where homelessness intersects with psychiatric morbidity, social exclusion, and a high demand for health and social services. Lisboa therefore represents a particularly relevant setting for studying longitudinal psychiatric trajectories in people experiencing homelessness, as also reflected in municipal strategic responses to homelessness and social exclusion.

These clinical challenges have led to specialized psychiatric approaches, including interstitial psychiatry, as well as to the establishment of Marontology. Marontology can be understood as a conceptual framework that addresses the complex psychiatric and medical comorbidities associated with homelessness, particularly in people with severe mental disorders, and that reflect the cumulative impact of chronic social exclusion, multimorbidity, and long-term instability (Gama Marques, 2021, 2023; Gama-Marques et al., 2025; Gama Marques & Bento, 2020; Gama Marques, Chesi et al., 2024; Lo et al., 2021; Spranger Forte et al., 2023).

### Aims of the Study

The longitudinal trajectories of psychiatric disorders among PEH are sparsely documented, especially in Portugal, where few studies track diagnostic changes or relate evolving clinical profiles to homelessness patterns, duration, and mortality.

This 20-year longitudinal study aimed to characterize the trajectories of PEH with psychiatric patients in Lisboa using demographic, clinical, service-use and mortality data. Specifically, the study had three objectives: (1) to describe the demographic and clinical characteristics of the general sample and their evolution over time; (2) to identify different duration-based cohorts of homelessness and compare their sociodemographic and clinical profiles; and (3) to examine clinical and survival-related predictors of the different cohorts. We hypothesized that longer durations of homelessness would be associated with greater clinical complexity, increased comorbidity, differentiated patterns of service use, and distinct mortality profiles across cohorts.

## Methods

### Procedure and Participants

The study included 333 psychiatric patients experiencing homelessness in the capital Lisboa, Portugal, followed over a period of 20 years. All patients attended at least one open group therapy session (OGT-PEH) at the psychiatric hospital in 2002. In 2020, participants were matched through hospital Electronic Health Record (EHR) identifiers, allowing longitudinal follow-up. Eligibility criteria included at least one confirmed psychiatric diagnosis according to the World Health Organization’s (WHO, 2016) International Classification of Diseases, 10th Edition (ICD-10) and documented episodes of homelessness (code Z59.X) during the study period. Patients with incomplete diagnostic or trajectory data were excluded from the analyses. Homelessness was classified in clinical records using *Fédération Européenne d’Associations Nationales Travaillant avec les Sans-Abri*’s (FEANTSA, 2007) ETHOS as rooflessness (sleeping rough) or houselessness (temporary accommodation). The dataset included both surviving and deceased individuals.

**Figure 1.**
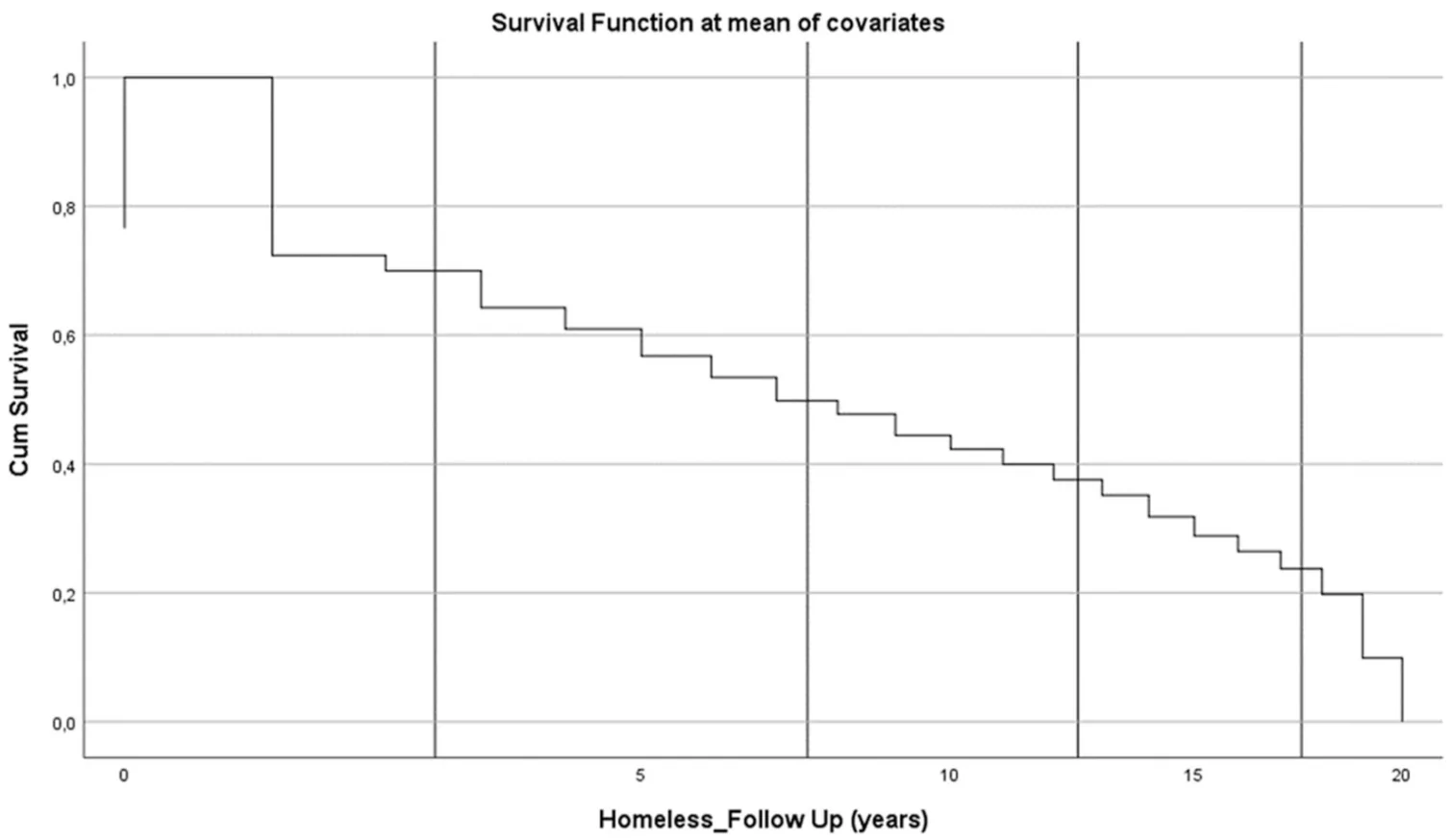
Cox regression survival function of homelessness in psychiatric patients over 20 years follow up. *Note*. The curve represents the probability of survival as a function of homelessness duration. Different declines in survival probability were used to establish cut-off points for cohort classification: Recent (⩽1 year), Early-stage (1–5 years), Prolonged (5–15 years) and Persistent (>15 years).

The duration of exposure to homelessness was defined as the total number of years between the first and last documented episodes of homelessness recorded in psychiatric contacts. Central to our investigation, four different homelessness trajectories were delineated using the survival curve function estimated from the Cox regression analysis (Figure 1): Recent homelessness (⩽1 year), Early-stage homelessness (1 –5 years), Prolonged homelessness (5 –15 years) and Persistent or chronic homelessness (>15 years). Observed inflexion points on the survival curve were used to determine empirically grounded cut-off points, supported by previous literature and the distribution of mortality risk in the follow-up. This methodological approach ensured that the four psychiatric cohorts experiencing homelessness were empirically substantiated, mutually exclusive, and collectively exhaustive.

This study was designed as a retrospective observational analysis using routinely collected clinical data. All data were fully anonymised prior to analysis and no direct contact with the participants occurred. In accordance with institutional regulations and standard practice for retrospective studies based on existing de-identified data, formal ethical review and informed consent were not required. The study did not involve experimental procedures and did not affect patient care.

### Variables and Data Sources

Data were extracted from EHR compiled over two decades. Sociodemographic variables included sex, age at initial and final psychiatric contact, birthplace, and marital status. Psychiatric diagnoses were recorded according to WHO ICD-10 criteria at both initial and final contact, allowing evaluation of diagnostic evolution over time. Lifetime comorbidities included intellectual disability (F70.X), personality disorders (F60.X), and alcohol or other substance abuse (F1X.X). Service-use variables included duration of psychiatric follow-up (years), number of psychiatric hospital admissions, and total length of inpatient stays (days). Survival-related variables included mortality status, age at death, and survival time. The four empirically defined cohorts served as the primary analytical framework, allowing comparisons of sociodemographic, clinical, service-use, and survival profiles across trajectories.

### Data Analysis

Cohorts were defined using Cox proportional hazards regression to model survival over a 20-year follow-up period. The cumulative survival function was examined to identify temporal patterns in mortality risk. Empirically derived cut-off points were established based on changes in the slope of the survival curve, allowing the classification of participants into four mutually exclusive and collectively exhaustive trajectory groups. Furthermore, examination of annual mortality rate rates identified distinct peaks in mortality risk at specified follow-up intervals.

We summarized the general sample and the four cohorts of PEH using descriptive statistics as means, standard deviations, medians, and interquartile ranges for continuous variables and frequencies or proportions for categorical variables. Group differences in continuous variables were tested with one-way analysis of variance (ANOVA) or Welch’s ANOVA, with Games–Howell *post hoc* tests for unequal variances and sample sizes. Effect sizes were reported as mean differences with 95% confidence intervals. Categorical variables were analyzed using Chi-square tests of independence or Fisher’s exact tests when expected cell counts were below five. Significant omnibus tests were followed by adjusted residual analyses with Bonferroni corrections for multiple comparisons.

Stepwise binomial logistic regressions using sociodemographic data, ICD-10 diagnoses, psychiatric comorbidities, and service-use variables were conducted to identify survival predictors and define cohorts. Model fit was assessed with Nagelkerke’s *R*^2^, and the classification performance with receiver operating characteristic (ROC) curve analyses that reported area under the curve (AUC), sensitivity, specificity, and overall precision. Predictive strength was quantified by odd ratios (ORs) with 95% confidence intervals (CIs). Given class imbalance, greater emphasis was placed on AUC and OR than on classification accuracy.

All analyses were performed using International Business Machines (IBM) Statistical Package for the Social Sciences (SPSS) (Version 26.0) and Jamovi(Version 2.3.28). A two-tailed significance level of *p* < .05 was applied in all tests.

## Results

### Data Summary

Initially, a summary statistics analysis was conducted for the overall sample, a dataset comprising 333 psychiatric patients from Lisboa’s PEH populations, observed over a 20-year period. Most of the patient cohorts experiencing homelessness were males (*n* = 292, 87.70%), with an average age of 47.16 years (*SD* = 12.29; M*dn* = 47.00; range: 18–75 years) at initial contact and 56.08 years (*SD* = 13.32; *Mdn* = 57.00; range: 18–85 years) at the final contact. The average duration of psychiatric follow-up was approximately 9 years (*SD* = 7.59; *Mdn* = 7.00; range: 0–20 years), during which patients underwent an average of 1.50 psychiatric hospitalizations (*SD* = 5.09; *Mdn* = 0; range: 0–81 times), with a mean inpatient stay of 51.25 days (*SD* = 370.58; *Mdn* = 0; range: 0–6350 days). For 45% of the sample, marital status was undetermined; most were single (30%) or married (15%), and fewer were divorced (8%) or widowed (2%).

At the point of initial contact, the distribution of psychiatric diagnoses among PEH patients was as follows: 41.10% had alcohol-related mental disorders (ICD-10 F10), 12.30% had unspecified personality disorders (ICD-10 F69), 9.90% had anxiety disorders (ICD-10 F41), 8.10% had unspecified psychosis (ICD-10 F29), 7.50% had schizophrenia (ICD-10 F20), and 5.70% had recurrent depressive disorder (ICD-10 F33). Diagnoses with less than 5% prevalence included opioid-related disorders (ICD-10 F11) and unspecified mental retardation (ICD-10 F79) at 4.80% each, persistent delusional disorders (ICD-10 F22) at 3.60%, organic psychosis (ICD-10 F06) at 1.50%, and vascular dementia (ICD-10 F01) at 0.60%, totaling 10%.

At the last interaction with PEH patients, psychiatric diagnoses were distributed as follows: ICD-10 F10 (alcohol-related disorders) constituted 34.80% of the cases, followed by ICD-10 F06 (organic psychosis) at 19.20%, ICD-10 F29 (unspecified psychosis) at 18.90%, ICD-10 F69 (unspecified personality disorders) at 8.10%, ICD-10 F41 (other anxiety disorders) at 6.30%, and ICD-10 F33 (recurrent depressive disorder) at 5.70%. Diagnoses with a prevalence below 5% comprised ICD-10 F11 (opioid-related disorders) at 3.30%, ICD-10 F79 (unspecified mental retardation) at 2.70%, and ICD-10 F25 (schizoaffective disorders) at 0.90%. Lifetime comorbidities comprise 54.70% for alcohol use, 7.80% associated with drug use, 17.40% for personality disorders, and 5.40% pertaining to intellectual disability.

The cohort exhibited a mortality rate of 31.20%, with a mean age at death of 61.06 years (*SD* = 11.78; *Mdn* = 61.00; range: 27–85 years), comprising 91.30% males, and 57.70% had a history of alcohol abuse. The average duration of psychiatric follow-up was 8 years (*SD* = 5.60; *Mdn* = 7.00; range: 0–20 years), with individuals experiencing a typical of 1.43 hospitalizations (*SD* = 2.70; *Mdn* = 0; range: 0–19 times) and a mean hospital stay of 87.68 days (*SD* = 622.58; *Mdn* = 0; range: 0–6350 days). The significant primary diagnoses included 43.30% with alcohol-related disorders (ICD-10 F10) and 14.40% with anxiety disorders (ICD-10 F41). At the final assessment, 37.50% had alcohol-related disorders (ICD-10 F10), 24% had organic psychosis (ICD-10 F06), and 14.40% had unspecified psychosis (ICD-10 F29).

Considering the entire dataset, the sample’s birthplaces were spread as follows: 25% originated from Lisboa, 28% from other regions within Portugal, 7% from Africa, and 1% from other European nations, India, and Brazil, with missing data accounting for 39%. In Lisboa, the main areas where the homeless sleep rough (the roofless) (Supplemental Figure 1) include the parishes of Arroios (18%), Misericórdia (14%), and Santa Maria Maior (10%), with other parishes accounting for 22%.

### Definition of Cohorts Based on Survival Analyses

The Cox Regression model survival analyses elucidated the homelessness trajectories by depicting the cumulative survival function over a 20-year follow-up period (Figure 1), thus illustrating gradual and significant declines at distinct intervals.

Four distinct cohorts were identified according to the duration of the homelessness. The Recent cohort (episodic) (up to 1 year) experienced significant survival decline in the first year. The Early-stage cohort (1–5 years) showed reduced survival up to year five. The Prolonged cohort (5–15 years) had a gradual decline in survival during mid-follow-up. The Persistent cohort (over more than 15 years) exhibited a measurable decline in long-term survival over 20 years. The survival function indicated a consistent decline over time, with notable drops early and midway, followed by a gradual decrease. Recent and Early-stage had steep early declines, Prolonged had mid-phase declines, and Persistent or chronic was prevalent among long-term survivors, maintaining stable survival after 15 years of homelessness.

### Mortality and Survival Outcomes

Mortality and survival outcomes were closely examined after defining cohorts. The Cox regression model (Figure 1) enabled the differentiation of trajectories, and further analyses provided information on the mortality distribution during follow-up. Specifically, annual mortality rates (Supplemental Figure 2) and survival trends between different groups were analyzed to determine whether the established thresholds aligned with the varying risk profiles.

The analysis of annual mortality rates (Supplemental Figure 2) further identified discrete peaks in mortality risk at specific follow-up intervals. The highest rates occurred during year 1 (64.29%), years 5 to 7 (57.14%–83.33%), and year 16 (62.50%). In contrast, substantial reductions were observed in years 8 (14.29%), 15 (20%), and particularly in the final years (⩽10 % in years 18–20). These findings indicate that mortality risk was concentrated at Recent phases, notably when moving from Recent to Early-stage, and from Early-stage to Prolonged trajectories, while patients who survived beyond 15 years (Persistent cohort) exhibited markedly lower mortality.

Joint analyses (Figure 1 and Supplemental Figure 2) reveal an uneven mortality distribution among the homelessness trajectories. Mortality peaks occur during the Recent phases (early and intermediate follow-up), with long-term survivors exhibiting greater resilience as survival stabilises after 15 years. Mortality risk is concentrated in transitions from Recent to Early-stage and Early-stage to Prolonged trajectories, while survivors beyond 15 years (Persistent cohort) have lower mortality. Early and intermediate cohorts (Recent, Early-stage, and Prolonged) show higher mortality and younger ages at death. In contrast, the Persistent cohort, despite increased psychiatric follow-up and hospitalizations, shows reduced mortality and older age at death. Individuals who experience homelessness beyond 18 years tend to show reduced mortality rates, suggesting a survivor effect among the chronically PEH with psychiatric diagnosis in Lisboa.

Mortality and survival outcomes demonstrate significant group proportions according to ICD-10 diagnosis at final contact and lifetime comorbidities, specifically for ICD-10 F06 (ꭕ^2^(3) = 14.38, *p* < .001), ICD-10 F10 (ꭕ^2^(3) = 20.53, *p* < .001), ICD-10 F29 (ꭕ^2^(3) = 8.47, *p* < .05), ICD-10 F79 (ꭕ^2^(2) = 9.00, *p* < .01), intellectual disability (ꭕ^2^(2) = 9.36, *p* < .01), and alcohol abuse (ꭕ^2^(3) = 31.24, *p* < .001). Mortality rates have significant major proportions related to the following: F06 – Prolonged (33%), Early-stage (23%); F10 – Prolonged (41%), Early-stage (31%); F29 – Early-stage (19%); F79 – Prolonged (5%); Intellectual disability – Prolonged (6%); Alcohol abuse – Prolonged (67%), Early-stage 12 (46%). The major factors influencing survival rates are significantly attributed to the following proportions: F06 – Persistent (27%); F10 – Recent (47%), Persistent (21%); F29 – Persistent (28%), Recent (18%); F79 – Persistent (7%); Intellectual disability – Persistent (15%); Alcohol abuse – Persistent (56%), Recent (48%). These results highlight the impact of specific diagnoses and comorbidities on mortality and survival rates in PEH cohorts.

### Analysis of Sociodemographic and Clinical Data in Patient Cohorts Experiencing Homelessness: Recent, Early-Stage, Prolonged, and Persistent

Table 1 presents an analysis of the clinical and sociodemographic characteristics of the four cohorts of PEH psychiatric patients. The cohorts predominantly consist of males, with percentages ranging from 83% to 92%. Significant variations in the average age of initial contact among the cohorts are particularly evident along with pronounced disparities in the age of final contact, with the Persistent group displaying considerable differences due to its lower mean age at first contact (*M* = 43.09 years) and higher mean age at last contact (*M* = 61.86 years), while the Recent cohort notably has the lowest mean age at final contact (*M* = 48.29 years). The cohorts differ greatly in terms of psychiatric follow-up and hospitalization, with Persistent having the longest follow-up and most admissions, Prolonged and Persistent experiencing lengthier and more frequent hospitalizations, and the Recent group showing shorter follow-up, fewer admissions, and shorter stays. Lifetime comorbidity rates vary with respect to alcohol and drug use, intellectual disability, and mortality. Prolonged shows elevated alcohol use, although no significant differences have been identified. The Persistent displays increased alcohol and drug abuse along with intellectual disabilities and has the oldest mean age of death at 67.29 years, which is not statistically different. While the incidence of personality disorders remains stable across different cohorts, the Persistent group stands out with a notably higher rate of 20.80%. The death rate is significantly elevated within the Prolonged and Early-stage cohorts, approximating 50%, whereas in the Persistent cohort, it is considerably lower at approximately 14%.

**Table 1.** Sociodemographic and Clinical Data of 333 Psychiatric Patients in Homelessness Cohorts, Post hoc (Games-Howell), Mean Comparisons, and Chi-square Analyses.

Homelessness cohorts	Recent (e)	Early-stage (t)	Prolonged (s)	Persistent (h)	Statistics
*N*	92	52	93	96	333 (total)
Gender, *n* (%)					*ns*
Male	81 (88.00)	45 (87.00)	86 (92.00)	80 (83.00)	
Female	11 (12.00)	7 (13.00)	7 (8.00)	16 (17.00)	
Age first contact, *M* years (±*SE*M), *SD; Mdn*; *IQR* (95% CI); (min, max)	48.04 (1.40), 13.43; 48.50; 21.00 (45.26, 50.83); (18.00, 75.00)	49.48 (1.84), 13.30; 50.00; 19.25 (45.78, 53.18); (23.00, 74.00)	49.18 (1.19), 11.51; 49.00; 17.00 (46.81, 51.55); (24.00, 71.00)	43.09 (1.06), 10.36; 43.50; 14.25 (45.26, 50.83); (18.00, 69.00)	*MD*(e-h) = 4.95* *MD*(t-h) = 6.39** *MD*(s-h) = 6.09***
Age last contact, *M* years (±*SE*M), *SD; Mdn*; *IQR* (95% CI) (min, max)	48.29 (1.40), 13.46; 49.00; 21.00 (45.41, 50.98); (18.00, 75.00)	53.08 (1.84), 13.25; 53.00; 20.00 (49.39, 56.77); (26.00, 78.00)	59.58 (1.22), 11.57; 60.00; 16.00 (57.16, 62.00); (30.00, 85.00)	61.86 (1.06), 10.35; 63.00; 14.25 (59.77, 63.96); (37.00, 85.00)	*MD*(e-s) = −11.38*** *MD*(t-s) = −6.50** *MD*(e-h) = −13.67*** *MD*(t-h) = −8.79***
Length psychiatric follow-up, *M* years (±*SE*M), *SD; Mdn*; *IQR* (95% CI); (min, max)	0.15 (0.04), 0.36; 0; 0 (0.08, 0.23); (0, 1.00)	3.60 (0.15), 1.05; 3.00; 2.00 (3.30, 3.89); (2.00, 5.00)	10.40 (0.31), 3.03; 10.00; 5.00 (9.77, 11.02); (6.00, 15.00)	18.77 (0.13), 1.25; 19.00; 2.00 (18.52, 19.02); (16.00, 20.00)	*MD*(e-t) = −3.44*** *MD*(e-s) = −10.25*** *MD*(t-s) = −6.80*** *MD*(e-h) = −18.62*** *MD*(t-h) = −15.17*** *MD*(s-h) = −8.37***
Admissions psychiatric hospitalizations, *M* (±*SE*M), *SD; Mdn*; *IQR* (95% CI); (min, max)	0.05 (0.04), 0.37; 0; 0 (−0.02, 0.13); (0, 3.00)	1.08 (0.30), 2.16; 0; 1.00 (0.48, 1.68); (0, 9.00)	2.38 (0.91), 8.74; 1.00; 2.00 (0.58, 4.18); (0, 81.00)	2.27 (0.33), 3.25; 0; 4.00 (1.61, 2.93); (0, 13.00)	*MD*(e-t)= −1.02** *MD*(e-s) = −2.32* *MD*(e-h) = −2.22*** *MD*(t-h) = −1.20*
Duration psychiatric hospitalizations, *M* days (±*SE*M), *SD; Mdn*; *IQR* (95% CI); (min, max)	0.39 (0.28), 2.68; 0; 0 (−0.16, 0.95); (0, 21.00)	142.52 (121.94), 879; 0; 8.25 (−102, 387); (0, 6,350)	58.42 (21.19), 204; 4.00; 30.00 (16.34, 100); (0, 1,578)	43.59 (13.40), 131; 0; 54.25 (16.99, 70.19); (0, 1,223)	*MD*(e-s) = −58.03* *MD*(e-h) = −43.20**
Lifetime comorbidities, *n* (%)					
Personality disorders (ICD-10 F69)	15_a_ (16.30)	8_a_ (15.40)	15_a_ (16.10)	20_a_ (20.80)	ꭕ^2^ = 1.09, *p* = .780
Use of alcohol	43_a_ (46.70)	28_a,b_ (53.80)	57_b_ (61.30)	55_a,b_ (57.30)	ꭕ^2^ = 4.26, *p* = .240
Use of drugs	2_a_ (2.20)	3_a,b_ (5.80)	6_a_ (6.50)	15_b_ (15.60)	Fisher = 11.70**
Intellectual disability (ICD-10 F79)	1_a_ (1.10)	0_a_ (0)	5_a,b_ (5.40)	12_b_ (12.50)	Fisher = 14.30***
Death, *n* (%)	15_a_ (16.30)	26_b_ (50.00)	49_b_ (52.69)	14_a_ (14.58)	ꭕ^2^ = 51.22***
Age death, *M* years (±*SE*M), *SD; Mdn*; *IQR* (95% CI); (min, max)	57.67 (2.99), 11.56; 61.00; 18.50 (51.26, 64.07); (40.00, 75.00)	58.42 (2.50), 12.72; 58.50; 17.50 (53.28, 63.56); (27.00, 78.00)	61.71 (1.74), 10.80; 60.00; 12.00 (58.61, 64.82); (29.00, 85.00)	67.29 (3.20), 11.97; 68.50; 16.50 (60.37, 74.20); (49.00, 85.00)	*ns*

*Note*.CI = confidence interval; F10 = alcohol-related disorders; F69 = unspecified personality disorders; F79 = unspecified mental retardation; ICD-10= International Classification of Diseases 1oth edition; IQR = interquartile range; M = mean; MD = mean difference; Mdn = median; min = minimum; max = maximum; ns = not significant; SD = standard deviation; SEM = standard error of the mean.

Each subscript letter denotes a subset of homeless cohorts whose column proportions do not differ significantly from each other at the .05 significance level.

* *p* < .05. **p < .01. ***p < .001.

**Figure 2.**
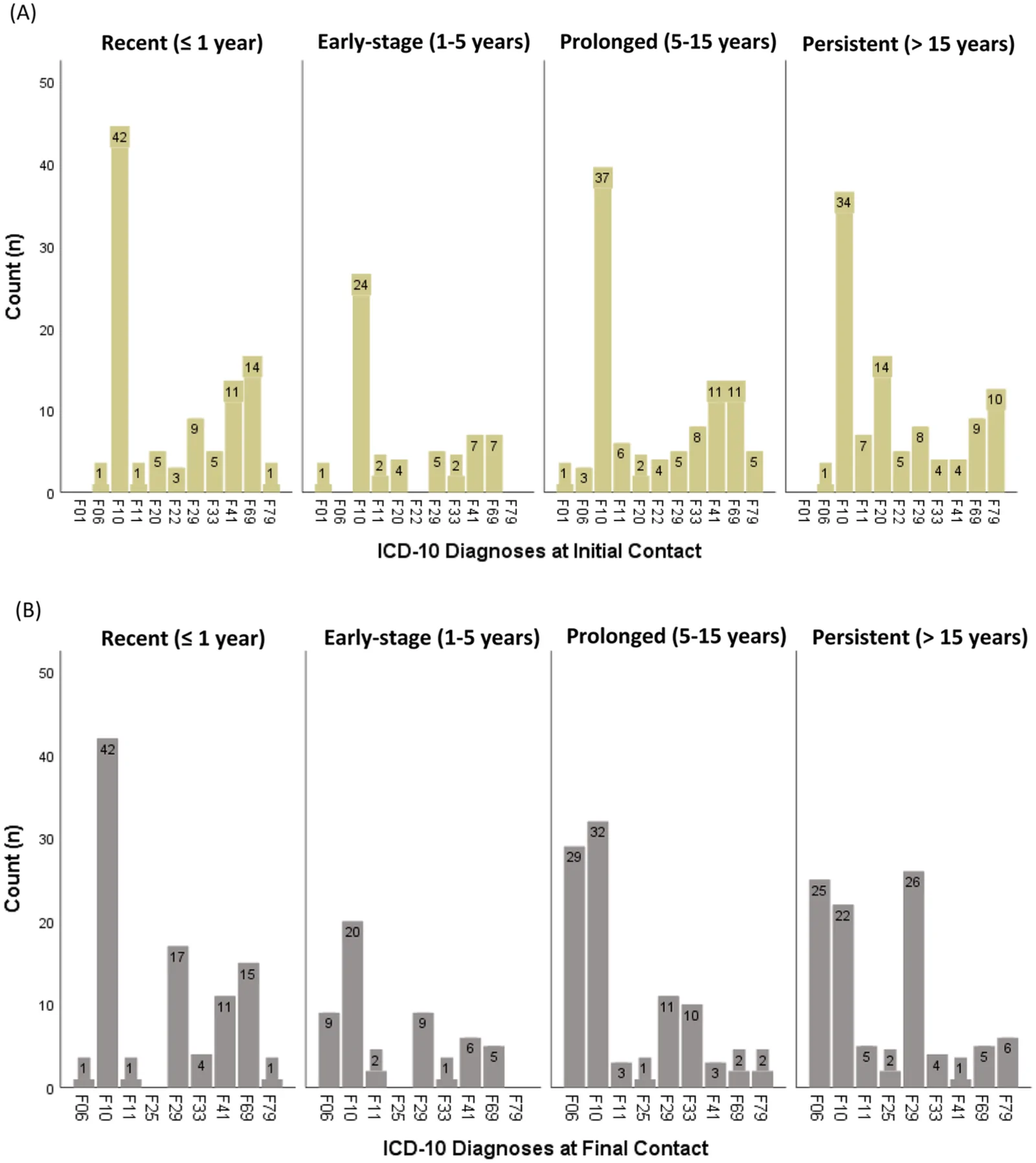
ICD-10 primary diagnoses in homelessness psychiatric cohorts: initial (a) and final contact (b).

Figure 2a shows the initial ICD-10 primary psychiatric diagnoses for the patient cohorts experiencing homelessness. ICD-10 F10 (alcohol-related disorders) is the most common at 41.10%, varying from 35.40% in Persistent to 46.2% in Early-stage, with no cohort differences. Significant variations were observed between the groups (χ^2^(30) = 46.03, *p* < .05). ICD-10 F11 (opioid-related disorders) increases from 1.10% in Recent to 7.30% in Persistent, and was more prevalent in Prolonged and Persistent. ICD-10 F20 (schizophrenia) peaks in Persistent (14.60%), while ICD-10 F41 (anxiety disorders) is lowest in Persistent (4.20%) compared to Early-stage (13.50%). ICD-10 F79 (unspecified mental retardation) is mainly in Persistent (10.40%) and Prolonged (5.40%), with low occurrences in other groups. The diagnoses for ICD-10 F06, F22, F29, F33, and F69 are consistent across cohorts. ICD-10 F69 (unspecified personality disorders) ranges from 15.20% in Recent, decreasing to 9.40% in Persistent. The data highlight distinct diagnostic patterns in PEH psychiatric cohorts, particularly ICD-10 F11, F20, F41, and F79, relating to common alcohol diagnoses (ICD-10 F10), outlining different clinical profiles within PEH groups. While alcohol-related disorders are ubiquitous, long-term chronic homelessness is more strongly associated with opioid use disorders, schizophrenia, and cognitive impairment, whereas less prolonged homelessness exhibits increased rates of anxiety and personality disorders.

Figure 2 (B) further delineates the ICD-10 primary psychiatric diagnoses recorded at the last contact. Significant variations between groups (χ^2^(24) = 81.91, *p* < .001). ICD-10 F06 (organic psychosis) was more prevalent in Prolonged (31.20%), Persistent (26%), and Early-stage (26%) than in Recent (1.10%). ICD-10 F10 (alcohol-related disorders) peaked in Recent (45.70%) over Persistent (22.90%), with Early-stage (38.50%) and Prolonged (34.40%) in between. ICD-10 F29 (unspecified psychosis) was most prevalent in Persistent (27.10%) compared to Prolonged (11.80%). ICD-10 F41 (anxiety disorders) was higher in Recent and Early-stage (12% each) than in Persistent (1%). ICD-10 F69 (unspecified personality disorders) were more prevalent in Recent (16.30%) than in Prolonged (2.20%), with Early-stage (9.60%) and Persistent (5.20%) in between. ICD-10 diagnoses F11, F33, F79, and F25 were low overall and evenly distributed, with a 6.30% occurrence of ICD-10 F79 (unspecified mental retardation) in Persistent. In general, Recent homelessness is associated with alcohol, anxiety, and personality disorders, while Persistent is more affected by psychosis and neurocognitive disorders. Early-stage and Prolonged show Recent profiles with overlapping features of Recent and chronic extremes.

### Hospital Admissions for ICD-10 Diagnoses

We analyze psychiatric hospitalization admissions for ICD-10 primary diagnoses at final contact among our four PEH cohorts (Supplemental Figure 3) using the Games-Howell post hoc test. In the Recent cohort, no significant admissions occurred; ICD-10 codes F29 and F10 had a slightly higher meaning (*M* = 0.12 and *M* = 0.07, respectively). Early-stage admissions were not significant, all under 2.60 times, with ICD-10 F06 and F33 at zero. Prolonged had the highest admission for ICD-10 F25 (*M* = 81.00; *n* = 1); significant hospitalizations were observed for ICD-10 F06 (*p* < .05) and ICD-10 F10 (*p* < .05) compared to ICD-10 F11, F69, and F79. The significant durations of Persistent were notable for ICD-10 F29 (*M* = 3.38, *p* < .01) and ICD-10 F06 (*M* = 3.36, *p* < .01); non-significant admissions were led by ICD-10 F25 (*M* = 7.00; *n* = 2), and the lowest were for ICD-10 F41, F69, and F79 at 0 times.

In general, psychiatric hospitalizations related to ICD-10 diagnoses exhibited variation. Individuals in the Recent cohort experienced slight increases in ICD-10 F29 (unspecified psychosis) and ICD-10 F10 (alcohol-related disorders), although overall admissions were insignificant. Admissions were low in the Early-stage cohort, with no cases of ICD-10 F06 (organic psychosis) or ICD-10 F33 (recurrent depressive disorder). The Prolonged group showed a notable increase in hospitalizations for ICD-10 F06 (organic psychosis) and ICD-10 F10 (alcohol-related disorders). The Persistent experienced more frequent hospitalizations for ICD-10 F29 (unspecified psychosis) and ICD-10 F06 (organic psychosis), whereas other diagnoses resulted in fewer or insignificant hospitalizations.

### Hospital Admissions for Sociodemographic Data and Lifetime Comorbidities

We analyze psychiatric hospital admissions based on sociodemographic data (sex, marital status) and lifetime comorbidities (alcohol and drug use, personality disorders) among four PEH cohorts using one-way ANOVA, Welch, and Games-Howell tests.

In the Recent cohort, no significance is found for sex (*F* = 0.26, *p* = .610), marital status (*F* = 2.37, *p* = .07), alcohol use (*F* = 2.24, *p* = .138), or personality disorders (*F* = 0.38, *p* = .541). Drug users have the highest admissions with *M* = 1.00 time (*F* (1,91) = 15.10, *p* < .001) (Supplemental Figure 4). In the Early-stage cohort, sex (*F* = 1.45, *p* = .246), marital status (*F* = 0.26, *p* = .610), drug use (*F* = 0.58, *p* = .451), and personality disorders (*F* = 0.08, *p* = .777) are not significant. Alcohol users have the highest admissions with *M* = 1.64 times (*F* (1,51) = 4.45, *p* < .05) (Supplemental Figure 4). In the Prolonged cohort, sex (*F* = 0.18, *p* = .676), marital status (*F* = 0.37, *p* = .830), alcohol use (*F* = 1.35, *p* = .250), and drug use (*F* = 0.09, *p* = .764) are not significant. Personality disorders result in the highest admissions, with *M* = 7.00 times (*F* (1,92) = 5.24, *p* < .01) (Supplemental Figure 4). In the Persistent cohort, sex (*F* = 0.05, *p* = .824), marital status (*F* = 0.90, *p* = .750), and alcohol use (*F* = 2.37, *p* = .07) are not significant. Individuals who consume alcohol have significantly higher admission rates, with a mean of 3.33 times (*F* (1,95) = 15.69, *p* < .001) (Supplemental Figure 4). Meanwhile, drug users exhibit the most substantial admissions, having a mean of 5.13 times (*F* (1,95) = 15.94, *p* < .001). Those with personality disorders follow with a mean of 4.40 times (*F* (1,95) = 12.09, *p* < .001) (Supplemental Figure 4).

In general, most hospital admissions to the Recent cohort were closely related to drug use, whereas admissions to the Early-stage cohort were primarily attributed to alcohol use. For the Prolonged cohort, admissions were predominantly associated with personality disorders, and the Persistent cohort saw significant admissions due to a combination of alcohol use, drug use, and personality disorders.

### Patient Psychiatric Cohorts Experiencing Homelessness: Predicting Survival Profiles

To predict survival *versus* mortality profiles of cohorts of PEH with psychiatric disorders (Recent, Early-stage, Prolonged, and Persistent), considering all clinical and sociodemographic variables, stepwise binomial logistic regressions were conducted (Table 2).

**Table 2.** Logistic Regression Models for Predicting Survival in Homelessness Psychiatric Cohorts.

Predictors	*B*	SE	Wald	*p*	OR [95% CI]	Nagelkerke *R*^2^
Recent cohort						.48
ICD-10 F29 (0)	1.48	0.64	5.34	<.01	4.40 [1.26, 15.40]	
ICD-10 F69 (0)	1.87	0.76	6.07	<.01	6.50 [1.47, 28.80]	
Use of alcohol (0)	1.80	0.44	16.70	<.001	6.04 [2.55, 14.33]	
Early-stage cohort						.14
Use of alcohol (0)	0.83	0.48	3.05	<.05	2.29 [1.00, 5.81]	
Persistent cohort						.46
ICD-10 F06 (0)	1.40	0.66	4.53	<.05	4.05 [1.12, 14.65]	
ICD-10 F29 (0)	1.63	0.63	6.62	<.01	5.11 [1.48, 17.69]	
Use of alcohol (1)	1.00	0.41	6.14	<.01	2.72 [1.23, 6.01]	

*Note. (0): absence; (1): presence; B* = logistic regression coefficient; CI = confidence interval; F06 = organic psychosis; F29 = unspecified psychosis; F69 = unspecified personality disorders; ICD-10 = International Classification of Diseases 1oth edition; OR = odds ratio; SE = standard error of the logistic regression coefficient

ICD-10 diagnoses are the final contact diagnoses retained in the models.

The Recent, Early-stage, and Persistent cohort models all showed statistical significance, with χ^2^(3) = 86.70, *p* < .001, explaining 48% of the variance for the Recent model, χ^2^(2) = 66.35, *p* < .05, explaining 14% of the variance for the Early-stage model, and χ^2^(3) = 92.51, *p* < .001, explaining 46% of the variance for the Persistent model, all according to Nagelkerke’s *R*^2^. No significant models was obtained for the Prolonged cohort.

The absence of ICD-10 F29 diagnoses (unspecified psychosis) increases survival odds by 4.40 in the Recent cohort and 5.11 in the Persistent cohort. In the Recent group, not having ICD-10 F69 (unspecified personality disorders) increased survival odds by 6.50. Non-use of alcohol improves survival odds in Recent (6.04) and Early-stage (2.29), while alcohol use increases it in Persistent (2.72), along with the absence of ICD-10 F06 (organic psychosis) at 4.05 odds. Overall, alcohol use and diagnoses of psychosis and personality disorders are strong survival predictors for the PEH with psychiatric disorders over 20 years.

### Cohorts of Homeless People With Psychiatric Disorders: Predictive Models

Predictive models identify subsets of PEH patients using clinical and sociodemographic variables (Table 1). Five binomial logistic regression models distinguish a specific PEH cohort from others, such as Recent, Early-stage, Prolonged, and Persistent (Table 3), assessed using ROC curve metrics (Supplemental Figure 5).

**Table 3.** Logistic Regression Models Predicting Homelesness Psychiatric Cohorts and ROC Curve.

Predictors	B	SE	*Z*	*p*	OR [95% CI]	ROC Curve			
						AUC	Se	Sp	Ac
Recent cohort						0.77	0.23	0.94	0.74
ICD-10 F41	2.83	0.60	4.70	<.001	16.94 [5.21, 55.10]				
ICD-10 F69	2.79	0.56	4.99	<.001	16.29 [5.45, 48.69]				
ICD-10 F10	2.13	0.44	4.82	<.001	8.39 [3.53, 19.94]				
ICD-10 F29	1.58	0.49	3.23	<.001	4.84 [1.86, 12.59]				
Survival (1–0)	1.14	0.33	3.42	<.001	3.11 [1.62, 5.97]				
Early-stage cohort						0.61	0.01	0.99	0.84
Death (1–0)	0.96	0.31	3.11	<.001	2.60 [1.42, 4.76]				
Prolonged cohort						0.73	0.20	0.95	0.73
ICD-10 F33	1.79	0.54	3.32	<.001	5.97 [2.08, 17.11]				
ICD-10 F06	1.36	0.36	3.82	<.001	3.90 [1.94, 7.86]				
Death (1–0)	1.30	0.27	4.82	<.001	3.66 [2.16, 6.20]				
ICD-10 F10	0.70	0.32	1.81	<.05	1.80 [1.00, 3.39]				
Early-stage + Prolonged						0.75	0.54	0.80	0.69
Death (1–0)	1.76	0.27	6.53	<.001	5.82 [3.43, 9.88]				
ICD-10 F20*	−1.75	0.63	−2.77	<.01	0.17 [0.05, 0.60]				
ICD-10 F06	0.87	0.32	2.71	<.01	2.39 [1.27, 4.47]				
Duration hospital	0.01	0.01	2.26	<.05	1.00 [1.00, 1.00]				
Persistent cohort						0.83	0.49	0.91	0.79
Drugs	2.26	0.53	4.27	<.001	9.62 [3.40, 27.22]				
Intellectual disability	2.24	0.66	3.40	<.001	9.38 [2.59, 34.02]				
Survival (1–0)	1.87	0.36	5.15	<.001	6.50 [3.19, 13.24]				
ICD-10 F29	0.79	0.36	2.21	<.01	2.20 [1.09, 4.41]				
Age (last contact)	0.10	0.01	6.50	<.001	1.09 [1.06, 1.12]				

*Note. Ac = accuracy; AUC = area under the curve; B* = logistic regression coefficient; Death: 1 = deceased, 0 = alive; F06 = organic psychosis; F10 = alcohol-related disorders; F20 = schizophrenia; F29 = unspecified psychosis; F33 = recurrent depressive disorder; F41 = anxiety disorders; F69 = unspecified personality disorders; ICD-10= International Classification of Diseases 1oth edition; *OR* = odds ratio; ROC = receiver operating characteristic; *SE* = standard error; Se = sensitivity; Sp = specificity; Survival: 1 = alive, 0 = deceased.

* The ICD-10 diagnoses are the final contact diagnoses retained in the models, except for ICD-10 F20*, which is the first contact diagnosis as predictor.

All models were statistically significant (*p* < .001), with variance explained ranging from 5% (Early-stage) to 37% (Persistent). Survival/mortality status was a significant predictor in all models. The Recent cohort model explained 25% of the variance (χ^2^(5) = 62.62, *p* < .001). The Early-stage model explained 5% (χ^2^(1) = 9.51, *p* < .001) and the Prolonged model explained 19% (χ^2^(4) = 47.37, *p* < .001). The combined Early-stage + Prolonged model for >1–15 years of chronic homelessness improved to 25% variance explained (χ^2^(4) = 70.02, *p* < .001). The Persistent model explained 37% of the variance (χ^2^(5) = 99.98, *p* < .001).

The Recent model shows disorder-specific risk with high odds of anxiety (F41; *OR* = 16.94) and personality disorders (F69; *OR* = 16.29), followed by alcohol-related disorders (F10; *OR* = 8.39) and unspecified psychosis (F29; *OR* = 4.84), and higher odds of survival (*OR* = 3.11). Its discrimination is acceptable (AUC = 0.77), but with low sensitivity and high specificity. The model explains 25% of the variance but misses many true positives at the threshold.

The Early-stage model retains mortality (*OR* = 2.60) as the sole predictor with poor discrimination (AUC = 0.61), very low sensitivity but higher specificity, resulting in minimal explained variance (5%), driven by class imbalance. The Prolonged model links depression (F33; *OR* = 5.97), organic disorders (F06; *OR* = 3.90), mortality (*OR* = 3.66), and alcohol consumption (F10; *OR* = 1.80). It shows moderate discrimination (AUC = 0.73), low sensitivity, high specificity, and explains 19% of the variance. Combining the Early-stage and Prolonged cohorts improves discrimination and classification through separate cohorts. These chronic combinations of PEH trajectories (>1–15 years of homelessness) show mortality (*OR* = 5.82) as a key predictor, with additional effects from organic disorders (F06; *OR* = 2.39) and length of hospital stay, while schizophrenia (F20) serves as a protective factor (*OR* = 0.17). Discrimination improves (AUC = 0.75), with a balance of sensitivity (0.54), specificity (0.80), precision (0.69), and 25% variance explained.

The Persistent cohort, characterised by the highest model performance with a good discriminative ability (AUC = 0.83) and 37% variance explained, includes substantive predictors such as drug use (*OR* = 9.62) and intellectual disability (*OR* = 9.38), with unspecified psychosis (F29; *OR* = 2.20) and older age linked to the trajectory. The Persistent cohort shows higher survival odds (*OR* = 6.50) than Early-stage and Prolonged mortality.

The results highlight cohort profiles that emphasise chronicity rather than mortality. The ROC profiles (Table 3; Supplemental Figure 5) show a preference for high specificity, risking the under-identification of true cases. Examining the Persistent and Recent groups, in addition to the unified Early-stage plus Prolonged cohort, shows significant differentiation, but the inherent variability in the Early-stage cohort cannot be fully captured by the predictors. The results of the predictive modeling reveal distinct clinical and sociodemographic patterns in homelessness trajectories, showing varying discriminatory efficacy between cohorts.

## Discussion

By examining longitudinal data from PEH with psychiatric diagnosis, the results reveal the complex clinical, social, and mortality-related dynamics that shape homelessness trajectories.

### Characterization of Patient Cohorts Experiencing Homelessness in Lisboa, Portugal

Addressing the first objective, the characterization of 333 PEH with psychiatric disorders followed over 20 years reveals an increasingly vulnerable and aging cohort. The predominance of single middle-aged men aligns with longstanding evidence that social fragmentation constitutes a core structural vulnerability (Bassuk et al., 1984; Bento & Barreto, 2002; Gama Marques, 2024). The increase in mean age from 47.16 to 56.08 years does not reflect clinical recovery but progressive aging within homelessness, demonstrating a systemic incapacity to support stable exits (Culhane et al., 2013). This chronicity places individuals at risk for accelerated aging processes, with associated multimorbidity, cognitive decline, and functional deterioration (Fazel et al., 2014; Hahn et al., 2006).

Clinically, the most common diagnoses were alcohol use disorders, personality disorders, mood disorders, and psychotic spectrum disorders, which are consistent with previous studies reporting a high prevalence of substance use, psychotic, mood and personality disorders among PEH (Barry et al., 2024; Fazel et al., 2008; Gama Marques et al., 2024; Monteiro Fernandes et al., 2022). The exceptionally high mortality rate (31.2%), with a mean age at death of 61.1 years, underscores alcohol use as a primary driver of premature mortality, consistent with studies demonstrating its strong association with organ failure, infections, trauma, and early death among PEH (Chang et al., 2023; Fazel et al., 2014). The rise in organic psychosis from 1.5% to 19.2% further suggests cumulative neuropsychiatric deterioration exacerbated by chronic alcohol use, untreated medical conditions, traumatic injuries, and the neurotoxic effects of sustained street exposure (Gama Marques, 2022b; Gutwinski et al., 2021; Pontes Silva & Gama Marques, 2023).

Comorbidity lifetime patterns, particularly those that involve alcohol, drugs, personality disorders, and intellectual disability, underscore a multidimensional clinical burden that complicates participation in treatment and long-term stabilisation (Ball et al., 2005; Timms & Drife, 2021). Healthcare utilisation patterns confirm this: while the mean number of hospitalisations was 1.5, some individuals accumulated more than 80 admissions and thousands of days of inpatient care, illustrating the well-documented revolving door dynamic among PEH (Canavan et al., 2012; Fonseca Barbosa & Gama Marques, 2023; Laliberté et al., 2019).

### Distinct Homelessness Trajectories

Regarding the second objective, the survival analysis identified four trajectories and revealed increased clinical complexity along with nonlinear mortality patterns.

The Recent cohort demonstrated acute vulnerability characterized by personality disorders, anxiety disorders, and alcohol misuse. These clinical features, associated with emotional dysregulation, impulsivity, and poor adherence to treatment, hinder stabilization and contribute to steep early mortality (Ball et al., 2005; Calvo et al., 2026; Morrison, 2009).

The Early-stage cohort (1–5 years) showed an increase in alcohol-related morbidity and marked mortality peaks, reflecting the destabilising effects of early homelessness, compounded by trauma, substance misuse, and limited-service access (Ball et al., 2005; Brown et al., 2022).

The Prolonged cohort (5–15 years) marked a shift toward neurocognitive deterioration, with increases in intellectual disability, organic psychosis, and polysubstance use, reflecting cumulative neurological harm and an established dependency on acute-care systems (Backer & Howard, 2007; Beijer & Andréasson, 2010; Fazel et al., 2014).

The Persistent cohort (>15 years) exhibited the most severe diagnostic profile but the lowest mortality, indicating a survivor effect in which long-term adaptation, street resilience, and recurrent hospitalizations create a maladaptive but stabilising equilibrium (Farrell, 2010; Gama Marques, 2022a; Grunberg & Eagle, 1990).

These trajectory-specific patterns are consistent with the broader literature showing that homelessness is not a homogeneous condition, but rather a spectrum of temporally and clinically differentiated pathways associated with varying levels of vulnerability, service use, and mortality risk (Kuhn & Culhane, 1998; McAllister et al., 2010, 2011).

### Predictors of Homelessness Trajectories

Toward the third objective, predictive modeling delineated distinct clinical, social, and survival profiles across homelessness trajectories. The Recent cohort was predicted by anxiety disorders, personality disorders, and alcohol misuse. These conditions, marked by emotional instability, interpersonal conflict, and impulsive decision-making, can precipitate acute crises that lead to homelessness (Kuhn & Culhane, 1998), and may also indicate a subgroup in which timely intervention could prevent longer-term embedding (Nilsson et al., 2019).

In contrast, the Early-stage and Prolonged cohorts demonstrated weak predictive specificity, reflecting clinical heterogeneity and shared vulnerability to early mortality. Their trajectories were defined not by distinctive clinical syndromes, but by cumulative physiological and psychological stressors intrinsic to homelessness (Morrison, 2009; Nilsson et al., 2018). Within these transitional phases, the absence of alcohol use emerged as a protective factor, underscoring alcohol’s role as an acute lethality driver during the early-to-mid cohort trajectory.

The Persistent cohort was predicted by intellectual disability and opioid use, indicating a profile of severe cognitive and functional dependence, and predisposing to further acquired brain injuries that perpetuate a cycle of disadvantage (Backer & Howard, 2007; Schütz, 2016). The paradoxical association of alcohol use with survival in this cohort reflects its role as an established coping mechanism within an already adapted chronic state (Calvo et al., 2026). Absence of organic psychosis predicted survival, making it a key survival factor, as its presence likely indicates severe neurological dysfunction. Collectively, these findings confirm a trajectory-dependent clinical ecology in which specific disorders shape vulnerability, entrenchment, and survival over time.

#### Clinical and Service Implications

The present findings have important implications for the organisation of medical psychiatric care and services. First, the marked mortality observed in recent and early-stage groups supports the need for early identification, rapid psychiatric assessment, and assertive outreach interventions targeting individuals at the beginning of homelessness trajectories. In these cohorts, alcohol-related disorders, anxiety, and personality pathology appear to be particularly relevant targets for prevention-orientated and treatment-focused interventions.

Second, prolonged and persistent patient’s cohorts highlight the need for long-term integrated models of care that combine psychiatry, addiction treatment, neurocognitive assessment, disability-informed support, and stable housing interventions. These findings support the relevance of multidisciplinary and public mental health approaches that go beyond the crisis response and address the cumulative effects of chronic homelessness, severe psychiatric disorders, and social exclusion.

Third, the high burden of hospital use among the longer-duration PEH cohorts suggests that service systems remain reactive rather than preventive. A more effective response would likely involve continuity of care, outreach psychiatry, sustained case management, and housing-linked mental health support aimed at reducing repeated hospitalisations and premature mortality.

#### Study Limitations, Strengths, and Future Research Directions

This study has important limitations. Retrospective design and reliance on clinical records limit generalisability and blur diagnostic trajectories. A relevant limitation is that the sample included only PEH with psychiatric disorders who had documented diagnoses and participated in a dedicated psychiatric, restricting the generalisability of the findings to the wider population of psychiatric disorders, particularly those without formal psychiatric follow-up or diagnosis. Defining homelessness through service contact can underestimate disengagement from care. In addition, causal relationships between psychiatric disorders and homelessness cannot be established. The sequences of exposure to shelter and street settings, as well as associations with specific subtypes of personality disorders, were not assessed.

The study also has important strengths. These include 20-year follow-up, the inclusion of both surviving and deceased individuals, and the use of survival analysis to derive empirically grounded duration-based groups. The study provides rare longitudinal evidence from Portugal in a clinically characterised population.

Future research should use prospective and mixed methods designs to examine clinical trajectories, treatment participation, and structural determinants of long-term homelessness. Future longitudinal and qualitative studies should examine psychosocial resilience, neurocognitive vulnerability, and structural determinants of long-term trajectories in psychiatric populations of PEH. Further work should evaluate prevention-orientated and public mental health interventions tailored to different trajectories, including early outreach, integrated mental health and housing models, and continuity of care for people with prolonged and persistent homelessness (Bhugra et al., 2024).

## Conclusions

This 20-year study shows that homelessness in psychiatric patients is dynamic, involving clinical decline, accelerated aging, and unequal risk. Alcohol use was a major cause of early death and diagnoses changed from behavioral disorders to organic psychosis, suggesting cumulative neuropsychiatric damage from long-term exposure to the street. Mortality peaked during trajectory transitions, while long-term chronic homelessness showed a survivor effect. The findings support precision psychiatry with early intervention, alcohol treatment, geriatric-informed care, and permanent supportive housing to reduce morbidity and premature mortality among PEH.

## Supplemental Material

sj-docx-1-isp-10.1177_00207640261468246 – Supplemental material for A 20-Year Longitudinal Study of Homelessness Among 333 Psychiatric Patients in Portugal: From Initial Episodes to Chronic Trajectories Supplemental material, sj-docx-1-isp-10.1177_00207640261468246 for A 20-Year Longitudinal Study of Homelessness Among 333 Psychiatric Patients in Portugal: From Initial Episodes to Chronic Trajectories by Joana Henriques-Calado, Nathalia Garcia and João Gama-Marques in International Journal of Social Psychiatry

sj-docx-2-isp-10.1177_00207640261468246 – Supplemental material for A 20-Year Longitudinal Study of Homelessness Among 333 Psychiatric Patients in Portugal: From Initial Episodes to Chronic Trajectories Supplemental material, sj-docx-2-isp-10.1177_00207640261468246 for A 20-Year Longitudinal Study of Homelessness Among 333 Psychiatric Patients in Portugal: From Initial Episodes to Chronic Trajectories by Joana Henriques-Calado, Nathalia Garcia and João Gama-Marques in International Journal of Social Psychiatry

sj-docx-3-isp-10.1177_00207640261468246 – Supplemental material for A 20-Year Longitudinal Study of Homelessness Among 333 Psychiatric Patients in Portugal: From Initial Episodes to Chronic Trajectories Supplemental material, sj-docx-3-isp-10.1177_00207640261468246 for A 20-Year Longitudinal Study of Homelessness Among 333 Psychiatric Patients in Portugal: From Initial Episodes to Chronic Trajectories by Joana Henriques-Calado, Nathalia Garcia and João Gama-Marques in International Journal of Social Psychiatry

sj-docx-4-isp-10.1177_00207640261468246 – Supplemental material for A 20-Year Longitudinal Study of Homelessness Among 333 Psychiatric Patients in Portugal: From Initial Episodes to Chronic Trajectories Supplemental material, sj-docx-4-isp-10.1177_00207640261468246 for A 20-Year Longitudinal Study of Homelessness Among 333 Psychiatric Patients in Portugal: From Initial Episodes to Chronic Trajectories by Joana Henriques-Calado, Nathalia Garcia and João Gama-Marques in International Journal of Social Psychiatry

sj-jpg-1-isp-10.1177_00207640261468246 – Supplemental material for A 20-Year Longitudinal Study of Homelessness Among 333 Psychiatric Patients in Portugal: From Initial Episodes to Chronic Trajectories Supplemental material, sj-jpg-1-isp-10.1177_00207640261468246 for A 20-Year Longitudinal Study of Homelessness Among 333 Psychiatric Patients in Portugal: From Initial Episodes to Chronic Trajectories by Joana Henriques-Calado, Nathalia Garcia and João Gama-Marques in International Journal of Social Psychiatry
