# Supplementary material for: A 20-Year Longitudinal Study of Homelessness Among 333 Psychiatric Patients in Portugal: From Initial Episodes to Chronic Trajectories
Source: Int J Soc Psychiatry. 2026 Jul 29;72(6):1485–98. doi: 10.1177/00207640261468246 (PMC13569911; doi:10.1177/00207640261468246)
Supplement: Supplementary file 1 [file sj-docx-1-isp-10.1177_00207640261468246.docx]

**Supplementary Figure 1**

*Map Displaying the Proportion of Rough Sleeping Areas for the Homeless in Lisboa's Parishes*
