# Supplementary material for: A 20-Year Longitudinal Study of Homelessness Among 333 Psychiatric Patients in Portugal: From Initial Episodes to Chronic Trajectories
Source: Int J Soc Psychiatry. 2026 Jul 29;72(6):1485–98. doi: 10.1177/00207640261468246 (PMC13569911; doi:10.1177/00207640261468246)
Supplement: Supplementary file 2 [file sj-docx-2-isp-10.1177_00207640261468246.docx]

**Supplementary Figure 2**
*Mortality Rate (%) of Homelessness Psychiatric Patients Over 20 Years Follow Up*

*Note.* The graph shows annual mortality rates based on psychiatric follow-up duration, peaking in years 1, 5-7, and 16, with major reductions in years 8, 15, and 18-20, indicating a survivor effect in long-term cohorts.
