# Supplementary material for: A 20-Year Longitudinal Study of Homelessness Among 333 Psychiatric Patients in Portugal: From Initial Episodes to Chronic Trajectories
Source: Int J Soc Psychiatry. 2026 Jul 29;72(6):1485–98. doi: 10.1177/00207640261468246 (PMC13569911; doi:10.1177/00207640261468246)
Supplement: Supplementary file 3 [file sj-docx-3-isp-10.1177_00207640261468246.docx]

**Supplementary Figure 3**

*Hospital Admissions for ICD-10 Diagnoses in Homelessness Psychiatric Cohorts*

**Persistent (> 15 years)**

**Prolonged (5-15 years)**

**Early-stage (1-5 years)**

**Recent (≤ 1 year)**
