# Supplementary material for: A 20-Year Longitudinal Study of Homelessness Among 333 Psychiatric Patients in Portugal: From Initial Episodes to Chronic Trajectories
Source: Int J Soc Psychiatry. 2026 Jul 29;72(6):1485–98. doi: 10.1177/00207640261468246 (PMC13569911; doi:10.1177/00207640261468246)
Supplement: Supplementary file 4 [file sj-docx-4-isp-10.1177_00207640261468246.docx]

**Supplementary Figure 4**

*Hospital Admissions for Lifetime Comorbidities in Homelessness Psychiatric Cohorts: Drug Use (A), Alcohol Use (B), and Personality Disorders (C)*

(A)

(B)

(C)

(C)

(C)
